# Lower Hemoglobin Concentration Is Associated with Retinal Ischemia and the Severity of Diabetic Retinopathy in Type 2 Diabetes

**DOI:** 10.1155/2016/3674946

**Published:** 2016-04-20

**Authors:** Alicia Traveset, Esther Rubinat, Emilio Ortega, Nuria Alcubierre, Beatriz Vazquez, Marta Hernández, Carmen Jurjo, Ramon Espinet, Juan Antonio Ezpeleta, Didac Mauricio

**Affiliations:** ^1^Department of Ophthalmology, University Hospital Arnau de Vilanova, 25198 Lleida, Spain; ^2^Department of Endocrinology and Nutrition, University Hospital Arnau de Vilanova, 25198 Lleida, Spain; ^3^Institut de Recerca Biomedica de Lleida, University of Lleida, 25198 Lleida, Spain; ^4^Department of Endocrinology and Nutrition, Institut d'Investigacions Biomediques August Pi Suñer, CIBER de Obesidad y Nutrición, Hospital Clinic, 08036 Barcelona, Spain; ^5^Department of Optometry, University Hospital Arnau de Vilanova, 25198 Lleida, Spain; ^6^Department of Endocrinology and Nutrition, CIBER of Diabetes and Associated Metabolic Diseases (CIBERDEM), Health Sciences Research Institute and University Hospital Germans Trias i Pujol, 08916 Badalona, Spain

## Abstract

*Aims*. To assess the association of blood oxygen-transport capacity variables with the prevalence of diabetic retinopathy (DR), retinal ischemia, and macular oedema in patients with type 2 diabetes mellitus (T2DM).* Methods*. Cross-sectional, case-control study (*N* = 312) with T2DM: 153 individuals with DR and 159 individuals with no DR. Participants were classified according to the severity of DR and the presence of retinal ischemia or macular oedema. Hematological variables were collected by standardized methods. Three logistic models were adjusted to ascertain the association between hematologic variables with the severity of DR and the presence of retinal ischemia or macular oedema.* Results.* Individuals with severe DR showed significantly lower hemoglobin, hematocrit, and erythrocyte levels compared with those with mild disease and in individuals with retinal ischemia and macular oedema compared with those without these disorders. Hemoglobin was the only factor that showed a significant inverse association with the severity of DR [beta-coefficient = −0.52, *P* value = 0.003] and retinal ischemia [beta-coefficient = −0.49, *P* value = 0.001]. Lower erythrocyte level showed a marginally significant association with macular oedema [beta-coefficient = −0.86, *P* value = 0.055].* Conclusions.* In patients with DR, low blood oxygen-transport capacity was associated with more severe DR and the presence of retinal ischemia. Low hemoglobin levels may have a key role in the development and progression of DR.

## 1. Introduction

Type 2 diabetes mellitus (DM) is a common disease with a prevalence and incidence that have increased in recent decades. The estimated increase in the total number of people with diabetes is from 366 million in 2011 to 552 million in 2030 [1]. Diabetic retinopathy (DR), including diabetic maculopathy, is a microvascular complication of DM and the leading cause of visual disability in adults of working age in developed countries. Its prevalence increases with age and the duration of diabetes. It is estimated that after 20 years of having diabetes, more than 20% of type 2 diabetic patients will have some degree of DR [2].

The initial changes in DR affect the microcirculation of the retina. The lesions observed in these patients are secondary to this microangiopathy and result in increased vascular permeability in the form of edema and retinal vascular occlusive disease. The retina is among the most active metabolic tissues of the human body and is highly sensitive to reductions in oxygen tension [3]. Some studies have suggested that hypoxia is a stimulus for the production of intraocular and systemic erythropoietin (EPO) [3–5] and for vascular endothelial growth factor (VEGF). Both EPO and VEGF are neuroprotective factors that possess likely angiogenic potential, leading to an increase in the proliferation of new retinal vessels and thus inducing the development of proliferative retinopathy.

Macular edema (ME) may appear at any stage of DR and is the main cause of central visual loss in diabetic patients. It is defined as an increase in tissue fluid, which causes a thickening of the retina and secondarily causes structural and functional alterations with important clinical consequences. According to data from the Wisconsin Epidemiologic Study of Diabetic Retinopathy (WESDR) [6], after 15 years of known diabetes, the prevalence of diabetic macular edema is approximately 25% and 14% in patients with type 2 DM treated with and without insulin, respectively.

Despite not fully understanding the pathogenic mechanisms of DR and ME, there are a large number of multicenter studies that have assessed the risk factors involved in DR and ME development and progression [7–11]. The duration of diabetes, glycemic control, and the systolic blood pressure are the most accepted and widely documented risk factors.

Several authors have also suggested that hemoglobin, hematocrit, and blood viscosity levels can contribute to the development and progression of DR [12–16]. Low hemoglobin was an independent baseline risk factor for the development of proliferative diabetic retinopathy (PDR) and severe visual loss in the Early Treatment Diabetic Retinopathy Study (ETDRS) report [12]. Other studies have corroborated this finding and found an improvement in DR status following a correction of the anemia [13, 17–19]. A prospective study published by Qiao et al. in 1997 showed an inverse independent relationship between hemoglobin concentrations and the development of DR, particularly severe forms of DR [13].

Similarly, we sought to assess whether a lower blood oxygen-transport capacity may be associated with the presence and severity of retinal disease in type 2 diabetes. To accomplish this, we determined the potential association of hematological factors, particularly the hemoglobin concentration, with the presence and severity of diabetic retinopathy, retinal ischemia, and macular edema in patients with type 2 diabetes.

## 2. Methods

### 2.1. Study Population

This was a prospective, observational, exploratory study of cases (*n* = 153) and controls (*n* = 159). It included subjects with type 2 DM with retinopathy (cases) and without DR (controls). This study was initially designed to evaluate the presence of subclinical micro- and macrovascular disease associated with diabetic retinopathy. In a recent publication, we described the increased frequency of subclinical atherosclerosis associated with diabetic retinopathy [20]. We took advantage of this study in which 312 type 2 diabetic patients aged 40–75 years without known clinical cardiovascular disease or renal failure (glomerular filtration rate < 60 mL/min) were included. The presence or absence of diabetic retinopathy was used for the allocation of the study groups by study design. To avoid including patients with additional microangiopathic burden, patients without retinopathy also had a current and previous normal urinary albumin: creatinine ratio (<30 mg/g). In the subgroup of diabetic patients with retinopathy, macroalbuminuria (urine albumin: creatinine ratio > 299 mg/g) was an additional exclusion criterion. We selected patients of several different ages to achieve a similar age distribution in the groups.

All of the diabetic patients were recruited from the outpatient clinic at our center. Potential candidates were identified from the screening and treatment diabetic eye disease program of our center. The clinical characterization of these patients has been recently described [20]. None of the patients included had a hemoglobinopathy. From an ophthalmological point of view, we also considered the following as exclusion criteria: refractive errors (spheric equivalent) > ±3.00 diopters; average opacity that made data analysis difficult; presence of other concomitant retinal pathology; surgery during the previous year; inflammatory conditions in the anterior or posterior segment; glaucoma treatment; and laser photocoagulation within the previous 6 months. The local ethics committee approved the study, according to the Helsinki Declaration. All patients signed an informed consent form.

### 2.2. Definition of Variables

All patients underwent a clinical evaluation that included age, sex, ethnicity, antidiabetic treatment, treatment for dyslipidemia and hypertension, use of antiplatelet drugs, systolic and diastolic blood pressure, body mass index, hemoglobin HbA1c, lipid profile, creatinine, and urinary albumin/creatinine ratio. Data on these variables from the study subjects have been already published [20] and are used here for the purpose of analysis of the present study outcomes. The methodology used to assess the clinical variables is described in detail elsewhere [20]. For the specific purpose of the current study, a blood count that included hemoglobin, hematocrit, and erythrocytes was conducted with the model TOASYSMEX XN-20 device (Roche, Japan).

### 2.3. Ophthalmic Examination

Patients underwent a complete eye examination to assess the presence or absence of diabetic retinopathy, clinically significant macular edema, and retinal ischemia.

According to a modified version of the American Academy Ophthalmology classification [21], the patients were classified according to the presence or absence of diabetic retinopathy (DR) and its severity: absence of DR (NDR), mild nonproliferative DR (NPDR), moderate to severe NPDR, and proliferative diabetic retinopathy (PDR). For the statistical analyses, we categorized the presence of diabetic retinopathy into 2 groups: group 1, mild DR, and group 2, DR > mild (including moderate DR, severe DR, or PDR).

Clinically significant macular edema (CSME) was defined according to the Early Treatment Diabetic Retinopathy Study (ETDRS) classification protocol: the presence of retinal thickening at or within 500 *μ*m of the center of the macula, hard exudates at or within 500 *μ*m of the center of the macula associated with thickening of the adjacent retina, and/or zones of retinal thickening 1 disc area in size and at least partially within a 1 disc diameter of the center [22].

The ophthalmologic examination included an automatic refraction (MRK-3100P, Huvitz), collection of the best corrected visual acuity, measurement of intraocular pressure (applanation tonometer measured by model AT 900, Haag-Streit), exploration with slit-lamp of the anterior segment, and pupil dilation with 1% tropicamide (Superfield NC and 90 D and +78 D Volk lens).

Visual acuity in each eye was measured on the Snellen chart and recorded as a decimal value with the best refraction for distance, and the data were applied to the logMAR (logarithm of the minimum angle of resolution) ETDRS chart. All data handling on visual acuity is expressed in logMAR format.

The fundi of all the patients were photographed using a 45° field stereoscopic digital fundus camera (TOPCON TRC: 50IX, retinal camera), centered first from the temporal to the macula and second from the nasal to the papilla [23]. For those who showed evidence of any retinopathy, additional 30° seven-field stereo digital pairs were taken.

The patients with identified diabetic retinopathy underwent a fluorescein angiography. The fluorescein angiography was performed according to a standard procedure and was used to assess the foveal avascular zone (FAZ) and peripheral retinal ischemia according to the ETDRS standards [24]. Two 30° fields extending along the horizontal meridian from 25° nasal to the disc to 20° temporal to the macula and four 45° peripheric fields were selected for further analysis. The presence of retinal ischemia was assessed by the capillary loss both in the peripheral areas and in the central subfield. The patients were classified into two groups: group 1, which had an absence of retinal ischemia, and group 2, in whom retinal ischemia was present (macular or peripheral to any degree).

 An optical coherence tomography (OCT) was carried out in all the patients to evaluate the macular area. The OCT used was the* Stratus Optical Tomography 3 (Carl Zeiss Meditec, Dublin, CA, USA).* The analysis of results was carried out using the* Fast Macular Thickness Map*.

### 2.4. Statistical Review

The continuous variables are presented as the mean and interquartile range, and the categorical variables are presented as proportions. We used the Mann-Whitney *U* and Chi-square tests to compare the means and the proportions of the risk factors for diabetic retinopathy and hematological factors (hemoglobin (Hb), hematocrit (Htc), erythrocytes, mean corpuscular volume (MCV), mean corpuscular hemoglobin (MCH), and mean corpuscular hemoglobin concentration (MCHC)), according to the presence of DR, the presence of mild DR versus DR > mild (moderate/severe/proliferative), the presence of retinal ischemia versus absence of retinal ischemia, and the presence of EMCS versus absence of EMCS.

Three logistic regression models for each of the variables considered were used (mild DR versus DR > mild, retinal ischemia, and EMCS). The variables that showed a statistically significant association in the bivariate analysis with each of the variables of interest were used as potential confounders. The adjustment of the logistic regression models followed the stepwise procedure, and those that showed a significant association with the variables of interest were adjusted. The saturated models mild DR versus DR > mild and retinal ischemia were adjusted by hemoglobin, sex, and the interaction sex-hemoglobin. Finally, the EMCS saturated model was adjusted by erythrocytes, sex, and interaction sex-erythrocytes. We tested whether there was an interaction between hemoglobin and sex for each of the variables of interest (mild DR versus DR > mild, the retinal ischemia, and the EMCS). A univariate logistic regression model was adjusted, in which the association was estimated and stratified by sex of the DR patient with hemoglobin for the variables that exhibited a significant interaction.

## 3. Results

### 3.1. Characteristics of Studied Subjects

As already explained above, the general clinical characteristics of the study groups have already been published [20]. The assessment of the characteristics of the study groups showed that, compared with the subjects without retinopathy, the diabetic subjects with retinopathy had a longer diabetes duration, higher HbA1c concentration, and higher prevalence of hypertension, systolic blood pressure, and urinary albumin excretion. The patients with retinopathy had also greater waist circumference and HDL-c. Additionally, they were more likely to be on insulin treatment and on antiplatelet drugs.

There were no differences in any of the hematological variables between patients with and those without retinopathy (Table 1). Additionally, no significant differences were observed between the concentrations of iron and ferritin (Table 1).

The distribution of retinopathy grades was as follows: 60 patients had mild NPDR (39.2%) and 93 patients NPDR > mild (57 moderate NPDR (37.3%), 29 severe NPDR (18.9%), and 7 PDR (4.6%)). The patients with retinopathy were slightly older. The proportion of male/female subjects was not different between the groups.

### 3.2. Relationship among Variables of Interest, General Factors, and Hematological Values in Patients with Diabetic Retinopathy

We next investigated the potential association of the hematological variables with the severity of retinopathy and the presence of retinal ischemia and macular edema in patients with DR.

The patients with a degree of diabetic retinopathy greater than mild (moderate/severe/proliferative) presented with higher glycated hemoglobin, systolic blood pressure, triglycerides, and urinary albumin excretion and were more likely on insulin treatment. Hb, Htc, and erythrocytes were significantly decreased with the increasing severity of DR.  Table 2 shows the variables that were statistically significant in the comparison between the groups.

Among the 153 study patients with DR, 58 had some degree of retinal ischemia (macular and/or peripheral). These patients were older, had a longer diabetes duration, were more likely to be on insulin treatment, and had a higher prevalence of microalbuminuria. The patients in the group without retinal ischemia were more often smokers, but this was not statistically significant (*P* = 0.0621). Hb, Htc, and erythrocytes were significantly reduced in the group with retinal ischemia compared with the group without retinal ischemia (Table 3).

In patients with clinically significant macular edema (*n* = 54), higher systolic blood pressure and urinary albumin excretion were observed. The levels of Hb, Htc, and erythrocytes were significantly reduced in this group compared with the patients without macular edema (Table 4).

### 3.3. Relation between DR, Retinal Ischemia, CSME, and Hematological Variables in Patients with Diabetic Retinopathy

The results of the multivariate models showed that lower levels of hemoglobin were associated with the severity of DR and the presence of retinal ischemia. In the case of macular edema, the association with the lower number of erythrocytes was significant. In addition, a significant interaction between the level of hemoglobin and sex, depending on the degree of diabetic retinopathy, was observed (Table 5). The multivariate analysis stratified by gender for the model of mild DR versus > mild DR yielded a significant hemoglobin and sex interaction, with higher levels of hemoglobin being against diabetic retinopathy in women [odds ratio (confidence interval 95%) = 0.374 (0.205–0.682); *P*: 0.001], whereas, in men, the odds ratio of this association was not significant.

## 4. Discussion

The results of our study failed to demonstrate an independent association between the concentration of Hb and the presence of DR. However, we found that lower Hb is associated with severe forms of DR and with the presence, until now not described, of retinal ischemia. We also demonstrated for the first time an association between low levels of red blood cells and the presence of EMCS.

Different factors have been described to be associated with the development and severity of DR in patients with type 2 DM [7–11]. These include the duration of DM, insulin treatment, high blood pressure, higher serum lipids, and high glycated hemoglobin urinary albumin excretion. Our study coincides with those published earlier and yields the results of a sample of patients without other advanced diabetic complications.

Anemia is suggested as another long-term complication of DM and, together with ischemia, has been associated with the development and progression of both microvascular and macrovascular complications. Ischemia is a critical component of DR, and it is potentially influenced by anemia, the main indicator of the blood oxygen delivery capacity [25]. Previous studies have suggested that, compared with normal red blood cells, the erythrocytes of diabetic patients have decreased deformability and increased aggregation at the capillary level [26–30]. This condition could make them more fragile and susceptible to breakage, which would lower the hemoglobin levels. This finding may suggest that hypoxia that occurs as a result of anemia acts as a stimulus for the release of inflammatory mediators and vasoproliferative factors, such as VEGF and EPO, that are capable of increasing vascular permeability and contribute to the development of ME and more severe forms of DR. High intravitreal levels of EPO have been shown in cases of proliferative DR and in patients with diabetic ME [3–5]^.^

There are several studies showing the role of anemia as an independent risk factor for diabetic retinopathy, particularly in population-based studies [12, 13, 15, 26]. Among them, Davis et al. [12] demonstrated that, in ETDRS, low hemoglobin and hematocrit levels were independent baseline risk factors for the development of high-risk proliferative diabetic retinopathy and severe vision loss over a 5-year follow-up. García-Ramírez et al. [3] found that diabetic patients with hemoglobin levels below 12 mg/dL were two times more likely to develop DR. A similar risk of anemia with severe retinopathy was also reported in a case series by Shorb [26]. Irace et al. [15] showed that the levels of blood viscosity, Htc, and hemoglobin were lower in patients with DR compared with subjects without retinopathy [15]. In addition, the levels of these hematological variables decreased as the degree of retinopathy increased. In that study, 190 type 2 diabetic patients both with and without DR were compared with 95 controls without DM. Premenopausal women and patients with renal failure were excluded. However, neither retinal ischemia nor macular edema was considered.

Other studies in the literature consider anemia to be a predictor of DR [11, 14, 17]. Rani et al. [17] showed that type 2 diabetic patients with anemia were 1.80 times more likely to develop diabetic retinopathy than were individuals without anemia. In contrast to our study, this article did not analyze a control group without retinopathy, nor was renal function accounted for. Karoli et al. [11] did consider a study population of type 2 diabetes with normoalbuminuria and normal glomerular filtration and established that low Hb levels are a risk factor for the development of DR. We did not find an association between low levels of Hb and the presence or absence of DR. This is most likely explained by the design of our study and the number of patients in the control group.

Anemia and low hematocrit have been found to also be associated with the presence of diabetic macular edema [31, 32]. In our study, we have corroborated these results, and we demonstrated an independent association between low levels of erythrocytes and the presence of EMCS.

There were several limitations in the present study that should be noted here because they may affect the generalization of our findings. Because this was a cross-sectional study, the results do not provide information on the potential causation effect of hemoglobin. Prospective and larger studies are required to overcome this limitation. Further, the results of this study should be interpreted with caution because it was not designed primarily to respond to these associations. The evaluation of the association between hematological parameters and the presence and severity of DR was a secondary objective. For this reason, though the majority of the patients included in the study were postmenopausal, this variable was not evaluated.

One of the strengths of this study is its case-control design, which enabled us to assess whether there are differences between patients with and without DR. The specific exclusion of other complications associated with diabetes, such as nephropathy and cardiovascular disease, provides us with a study population that is free of microvascular and macrovascular disease, with the exception of the retinopathy. This eliminates the influence of these factors, especially renal insufficiency, as confounding variables and differentiates this study from the majority of the previously published studies.

## 5. Conclusion

There is an association of low blood oxygen-transport capacity, measured by hemoglobin concentration, and an increased severity of DR and the presence of retinal ischemia. These findings might have clinical implications, such as those in attempt to better control hemoglobin concentrations in diabetic patients, particularly in those with advanced forms or DR ischemia. In patients with these conditions, it would be advisable to monitor and prevent anemia. Prospective studies specifically designed to test these associations are needed to confirm low values of hemoglobin as a risk factor for DR and retinal ischemia also in patients with type 1 DM. Clinical trials in patients with anemia are needed for the further confirmation of these associations.

## Figures and Tables

**Table 1:** Hematological variables of the study groups according to presence or absence of diabetic retinopathy (DR).

	Without DR (*n* = 159)	With DR (*n* = 153)	*P* value
Leukocytes (×10^9^/L)	6670 (5600–7730)	6795 (5685–8035)	0.3765
Hemoglobin (gr/dL)	13.8 (13–14.6)	13.6 (12.8–14.8)	0.5435
Erythrocytes (×10^12^/L)	4.6 [4.4–5.0]	4.6 [4.4–4.9]	0.9407
Hematocrit (%)	41.10 [39–43.9]	40.95 [38.8–43.25]	0.7114
MCV (fL)	87.8 [85.1–90.5]	87.6 [85.0–90.1]	0.6169
MCH (pg)	29.4 [28.4–30.6]	29.3 [28.0–30.7]	0.4814
MCHC (g/dL)	33.5 [32.8–34.1]	33.4 [32.7–34.3]	0.7543
Fe (*μ*g/dL)	78 (63–99)	74 (58–96)	0.2534
Ferritin (ng/mL)	131.2 (55.5–227.8)	98.5 (38–211.9)	0.0624

Data are presented as medians and interquartile ranges. BP: blood pressure, MCV: mean corpuscular volume, MCH: mean corpuscular hemoglobin, and MCHC: mean corpuscular hemoglobin concentration.

**Table 2:** General and hematological variables that showed statistically significant differences in the comparison between groups according to severity of retinopathy in the group of patients with diabetic retinopathy.

	Mild diabetic retinopathy (*n* = 60)	>mild diabetic retinopathy (*n* = 93)	*P* value
Serum glucose (mg/dL)	150 [113–181]	175 [135–217]	0.012
Systolic BP (mm Hg)	139 (125–147)	149 (135–164)	0.0008
Insulin treatment, *n* (%)	2 (13.88%)	16 (10.45%)	0.0020
Triglycerides (mmol/L)	138 [88–177]	110 [81–153]	0.041
Urinary albumin: creatinine ratio, mg/g	8.21 [4.84–18.8]	18.7 [7.3–39.6]	0.003
Leukocytes (×10^9^/L)	7.40 [6.11–8.39]	6.42 [5.54–7.84]	0.050
Hemoglobin (gr/dL)	14.0 [13.2–15.0]	13.4 [12.6–14.4]	0.005
Erythrocytes (×10^12^/L)	4.8 [4.6–5.0]	4.6 [4.4–4.9]	0.007
Hematocrit (%)	42.3 [40.0–43.8]	40.4 [38.4–42.1]	0.005

**Table 3:** General and hematological variables that showed statistically significant differences in the comparison between groups according to the presence or absence of retinal ischemia in the group of patients with diabetic retinopathy.

	Absent retinal ischemia (*n* = 95)	Present retinal ischemia (*n* = 58)	*P* value
Urinary albumin: creatinine ratio, mg/g	9.4 [5.2–21.4]	25.0 [8.4–76.2]	0.002
Disease duration (yr)	10 (5–15)	16.5 (8–23)	0.0015
Insulin treatment, *n* (%)	40 (42.1%)	43 (74%)	0.038
Smoking	7 (12.1%)	23 (24.5%)	0.0621
Hemoglobin (gr/dL)	14.0 [13.1–14.9]	13.1 [12.5–13.9]	<0.001
Erythrocytes (×10^12^/L)	4.8 [4.5–5.0]	4.6 [4.3–4.9]	0.002
Hematocrit (%)	41.7 [39.9–43.8]	39.7 [36.6–41.4]	<0.001

**Table 4:** General and hematological variables that showed statistically significant differences in the comparison between groups according to the presence or absence of CSME in the group patients with diabetic retinopathy.

	Diabetic retinopathy without CSME (*n* = 99)	Diabetic retinopathy with CSME (*n* = 54)	*P* value
Urinary albumin: creatinine ratio, mg/g	9.2 [5.1–22.0]	23.3 [9.4–68.8]	<0.001
Systolic BP (mm Hg)	141 (129–154)	148 (135–165)	0.0337
Hemoglobin (gr/dL)	13.8 [13.0–14.9]	13.2 [12.6–14.1]	0.047
Erythrocytes (×10^12^/L)	4.7 [4.5–5.0]	4.6 [4.3–4.8]	0.019
Hematocrit (%)	41.3 [39.2–43.5]	40.3 [38.3–42.0]	0.047

**Table 5:** Association between hematologic variables and mild DR versus > mild DR, ischemia versus no ischemia, and CSME versus no CSME.

	Mild DR versus > mild DR				Ischemia				CSME			
	Model 1		Model 2		Model 1		Model 2		Model 1		Model 2	
	Coefficient	*P* value	Coefficient	*P* value	Coefficient	*P* value	Coefficient	*P* value	Coefficient	*P* value	Coefficient	*P* value
Hemoglobin	−0.36	0.005	−0.52	0.003	−0.42	0.004	−0.49	0.001	—	—	—	—
DM duration	—	—	—	—	0.04	0.038	—	—	—	—	—	—
Sex	—	—	6.31	0.009	—	—	1.19	0.565	—	—	−2.40	0.254
Sex *∗* hemoglobin	—	—	−0.46	0.009	—	—	−0.09	0.554	—	—	—	—
Erythrocytes	—	—	—	—	—	—	—	—	−0.93	0.030	−0.86	0.055
Sex *∗* erythrocytes	—	—	—	—	—	—	—	—	—	—	0.48	0.284

Models mutually adjusted.
